# Accurate Measurement of Vitamins in Foods and Tissues

**DOI:** 10.6028/jres.093.077

**Published:** 1988-06-01

**Authors:** J. N. Thompson

**Affiliations:** Nutrition Research Division, Health Protection Branch, Tunney’s Pasture, Ottawa, Ontario, Canada K1A OL2

Dramatic advances in the development of techniques and instruments in recent decades have improved the quality of all analytical work including that directed at determining vitamins. The pursuit of accuracy, however, still involves detailed examination of every step in a procedure, including studies on seemingly trivial manipulations. New methods must also be checked against established procedures and finally, tested in collaborative studies. The development and evaluation of methods for measuring vitamin A in Canada is described as an example. These procedures were designed to replace the antimony trichloride color reaction in the analysis of various materials ranging from serum collected in nutrition surveys to milk being checked for fortification.

Several publications have recommended simple fluorometric methods for the measurement of vitamin A in blood. Comparison of the results of fluorometric and colorimetric methods, however, revealed large differences. Investigations confirmed that blood contains at least one fluorescent carotenoid, phytofluene, which interferes in analysis for vitamin A [1]. Chromatographic purification or correction formulas were needed to eliminate the errors [2,3].

Milk, in contrast, contains little phytofluene. Fatty acids from milk include fluorescent lipids, but these can be removed by saponification. A simple method was developed for milk in which 1 mL was saponified and then extracted with hexane in a stoppered centrifuge tube [4]. The method could be used to examine large numbers of samples and it was adopted by several laboratories involved in milk analysis. Uniformity in milk analysis was maintained in Canada by a quality assurance program. Between 1979 and 1982, ten laboratories in Canada participated in a project in which 4 samples of milk were circulated for analysis on 20 occasions. The standard deviations of 6 laboratories using the fluorometric method were usually less than 10%.

Liquid chromatography (LC) methods are now often recommended for the analysis of vitamins and there are many reports of the separation and detection of retinol, retinol esters and carotenoids. Methods have been developed for normal and reversed phase separations of retinol after saponification of retinol esters extracted directly from foods and tissues [5]. Retinol and carotenoids are often measured in extracts of serum by reversed-phase LC [6]. Unfortunately, collaborative tests of LC methods for measuring retinol in foods have yielded disappointingly high standard deviations, and few of the many LC methods proposed for the measurement of vitamin A in blood have been examined critically or collaboratively.

To assist the evaluation and development of methods for vitamin A, a reference LC method was designed for the analysis of foods and tissues. The method involved saponification of substantial amounts of sample in ethanolic pyrogallol. A small portion of the digest was extracted in centrifuge tubes, and the extract was evaporated under nitrogen in the presence of a trace of nonvolatile hexadecane. The residue was subjected to LC on a silica column. All-trans retinol was eluted after 5 minutes and small amounts of 13-cis isomer were eluted a minute earlier. Each step in the method was studied in detail and modifications were introduced to eliminate possible sources of error. The tests included comparisons of various methods of saponification, and investigations of distribution ratios for retinol between solvents and saponification digests. The stability of retinol was investigated during evaporation of solutions in common solvents. Two important points emerged. First, the vitamin was rapidly decomposed in chlorinated solvents; this decomposition was due to the presence of HCl and it was eliminated when the solvent was washed with alkali or water. Second, the vitamin was unstable after pure solutions in any volatile solvent were evaporated because it was then deposited alone on the inside surface of a dry container where it was difficult to protect, even with nitrogen. The survival of vitamin in many methods of analysis thus depends on poorly understood roles of protective materials in extracts which are derived accidentally from reagents or samples.

The tendency of retinol to decompose in air affects the accuracy of standards. Retinol purchased from suppliers typically contains more than 20% impurities and once a vial is opened, the contents deteriorate rapidly. Solutions of pure retinol could be prepared by collecting and pooling eluate from several LC runs, and the concentrations could be determined accurately from the absorbance. Although fresh preparations yielded excellent standards for LC, there appeared to be no reliable method of storing them for longer than a few days. Solutions in organic solvents decomposed when sealed under nitrogen, and exposure to traces of air accelerated the process. In contrast, solutions of retinol in cottonseed oil, which can be quantitatively diluted in solvent for use in LC, were relatively stable and their vitamin content fell less than 1% per month. Oil solutions were therefore prepared for use as standards in routine analysis, and they were calibrated at intervals by LC analysis using freshly purified retinol as a primary standard.

A Waters 990 photodiode array detector, which scans a range of wavelengths during LC and plots complete spectra of the eluate, was used to test for the presence of interfering substances. The detector confirmed that the main peak in the LC analysis for vitamin A in all samples was all-trans retinol. Moreover, the spectrum of the smaller, earlier peak was similar to that of the 13-cis isomer when extracts were prepared from infant formula or milk. The occurrence of the cis isomers was not confirmed in blood, however, and the small peak detected by the fixed wavelength detector at the retention time of the 13-cis isomer was concluded to be a polar carotenoid.

Adjustments of the saponification step permitted the reference method to be applied to oils, foods and feeds. Comparisons of the reference method with the fluorometric method indicated that the latter overestimated the true retinol content of unflavored milk by less than 10%.
